# Serum-Based Protein Biomarkers in Blast-Induced Traumatic Brain Injury Spectrum Disorder

**DOI:** 10.3389/fneur.2012.00107

**Published:** 2012-07-06

**Authors:** Denes V. Agoston, Mohammad Elsayed

**Affiliations:** ^1^Department of Anatomy, Physiology and Genetics, Uniformed Services UniversityBethesda, MD, USA; ^2^Experimental Trauma Unit, Department of Neuroscience, Karolinska InstitutetStockholm, Sweden; ^3^Veterans Affairs Central Office, U.S. Department of Veterans AffairsWashington, DC, USA

**Keywords:** blood, proteomics, traumatic, brain, injury, blast, biomarkers, serum

## Abstract

The biological consequences of exposure to explosive blast are extremely complex. Serum protein biomarkers in blast-induced traumatic brain injury (bTBI) can aid in determining injury severity, monitoring progress, and predicting outcome. Exposure to blast results in varying degrees of physical injury. Explosive blast can also induce psychological stress that can contribute to or amplify the extent of physical damage. Given the complexity, scale of injury, and variety of symptoms, bTBI may be best described as a spectrum disorder. In this focused review, we summarize the status of serum protein biomarkers in bTBI in the context of the classification and pathological changes of other forms of TBI. Finally, we recommend specific and easily implementable measures to accelerate serum protein biomarker discovery and validation in bTBI.

Serum protein biomarkers have long held promise in the treatment of traumatic brain injury (TBI). They can aid in diagnosing the disease, monitoring progress, predicting outcome, and providing pertinent molecular information about ongoing pathological changes for designing evidence-based therapeutic interventions (Kochanek et al., 2011). Serum protein biomarkers are of special importance in blast-induced TBI (bTBI) because they are typically associated with military operations with limited access to imaging and other diagnostic tools of hospitals (Agoston et al., 2009).

The physical and biological consequences of explosive blast are extremely complex. Blast generates high energy supersonic pressure waves, heat, toxic gases, electromagnetic pulses, etc. (Champion et al., 2009;Ramasamy et al., 2009a,b; Hicks et al., 2010; Nakagawa et al., 2011). How each of these forces, separately or in a combinatorial fashion interact with the brain and body is still poorly understood. While the cause of bTBI is exposure to blast, injury severity may range from mild to severe and result in outcomes that cover a wide set of symptoms (Mayorga, 1997; Guy et al., 2000; Elder and Cristian, 2009; Elder et al., 2010; Rosenfeld and Ford, 2010; Marion et al., 2011). Exposure to blast can also cause severe psychological stress that can contribute to or amplify the extent of physical damage (Kluger et al., 2004; Ling et al., 2009; Wallace, 2009; Wolf et al., 2009; Ling and Ecklund, 2011). Accordingly, bTBI may be best described as a spectrum disorder. Similar to other forms of TBI, the classification of bTBI is currently based on subjective neurobehavioral evaluations including the Glasgow Coma Scale (GCS) and the Military Acute Concussion Evaluation (MACE; Cernak et al., 1999; Secer et al., 2007; Bochicchio et al., 2008; Peleg and Savitsky, 2009; Rosenfeld and Ford, 2010; Tarmey et al., 2011). These functional assessments are only occasionally supplemented with information from neuroimaging techniques, such as computed tomography (CT) and magnetic resonance imaging (MRI; Ling and Ecklund, 2011).

In this paper, we provide a brief overview of the status of serum protein biomarkers in bTBI. Because of the limited information about protein biomarkers specific to blast injury, we will discuss them in the context of the classification and pathological changes of other forms of TBI.

## Epidemiology of TBI and bTBI

Traumatic brain injury is an enormous public health concern. The Centers for Disease Control and Prevention (CDC) estimates that ∼1.7 million Americans sustain TBI every year (Coronado et al., 2011). TBI also contributes to about a third of all injury-related deaths, resulting in over 52,000 deaths a year (Coronado et al., 2011).

Improving TBI treatment for military personnel is especially pressing. The incidence of TBI for armed forces, even during peacetime, is greater than civilian populations (Ommaya et al., 1996). Also, due in part to the nature of modern combat, the incidence rates of TBI have been increasing since 2000 (Sayer, 2012). Although it is difficult to assess exactly how many soldiers are victims of TBI (especially because many are not properly diagnosed), estimates from Veterans Affairs medical records report that about 7% of veterans from Iraq and Afghanistan received a TBI diagnosis (Taylor et al., 2012). Other survey studies suggest that 11–23% of military personnel deployed to Iraq or Afghanistan may have sustained at least mild TBI (Sayer, 2012). Studies from the Rand Corporation estimate that about of fifth of returning members suffer from TBI (Tenielian and Jaycox, 2008).

Blast injury from improvised explosive devices (IEDs) is an especially common form of TBI among military populations. According to the Joint Theater Trauma Registry, IEDs were the source of about 80% of all casualties of veterans from Iraq and Afghanistan between October 2001 and January 2005 (Owens et al., 2008). In another review of US Army casualties in Afghanistan and Iraq between 2001 and 2007, explosions were linked with 63% of all TBI diagnoses (Wojcik et al., 2010). Another similar survey of US Navy and Marine casualties in Iraq in 2004 found that 52% of all TBI cases involved explosions (Galarneau et al., 2008). Despite the variability of these studies, it is evident that TBI and bTBI especially affects a great deal of soldiers, thus requiring the need for improved diagnostics and treatments.

## Classification of Blast-Induced Traumatic Brain Injury

**Figure 1:**
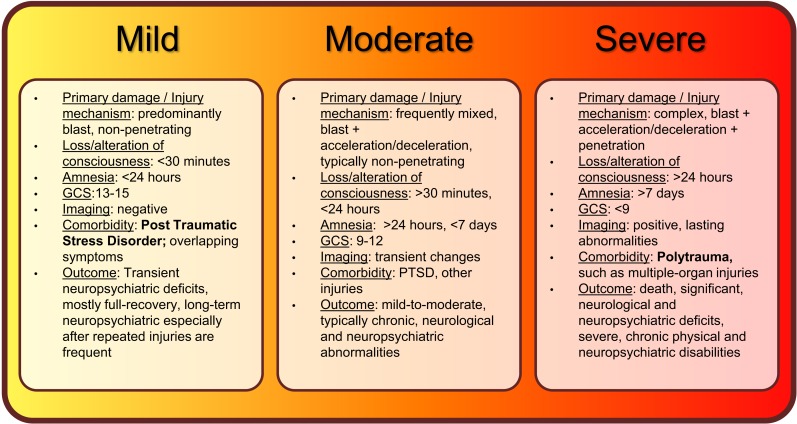
**Summary of current classifications, injury mechanisms, clinical symptoms, and outcomes of bTBI spectrum disorder**.

**Figure 2:**
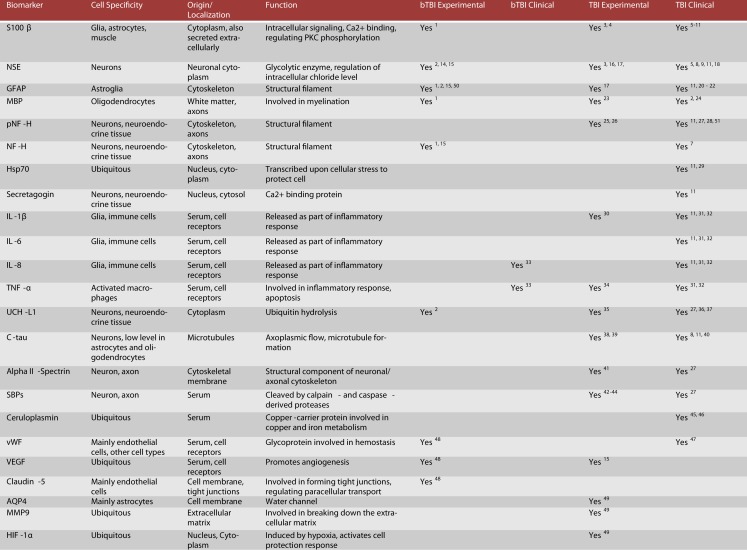
**Candidate protein biomarkers for blood-based diagnostics in traumatic brain injury**. ^1^Gyorgy et al. (2011), ^2^Svetlov et al. (2010), ^3^Hardemark et al. (1989), ^4^Rothoerl et al. (2000), ^5^Bellander et al. (2011), ^6^Gonzclez-Mao et al. (2011), ^7^Haqqani et al. (2007), ^8^Begaz et al. (2006), ^9^Pleines et al. (2001), ^10^Townend et al. (2006), ^11^Zurek and Fedora (2012), ^12^Honda et al. (2010), ^13^Berger et al. (2005), ^14^Cheng et al. (2010), ^15^Kwon et al. (2011), ^16^Pineda et al. (2004), ^17^Woertgen et al. (2002), ^18^Graham et al. (2011), ^19^Hergenroeder et al. (2008), ^20^Papa et al. (2012), ^21^Vos et al. (2010), ^22^Pelinka et al. (2004), ^23^Liu et al. (2006), ^24^Berger (2006), ^25^Petzold (2005), ^26^Anderson et al. (2008), ^27^Siman et al. (2009), ^28^Sandler et al. (2010), ^29^da Rocha et al. (2005), ^30^Kinoshita et al. (2002), ^31^Stein et al. (2011), ^32^Hayakata et al. (2004), ^33^Surbatovic et al. (2007), ^34^Vitarbo et al. (2004), ^35^Liu et al. (2010), ^36^Berger et al. (2012), ^37^Papa et al. (2010), ^38^Zemlan et al. (2002), ^39^Gabbita et al. (2005), ^40^Bulut et al. (2006), ^41^Pike et al. (2001), ^42^Newcomb et al. (1997), ^43^Ringger et al. (2004), ^44^Saatman et al. (2010), ^45^Dash et al. (2010), ^46^Young et al. (1988), ^47^De Oliveira et al. (2007), ^48^Ahmed et al. (in preparation), ^49^Higashida et al. (2011), ^50^Garman et al. (2011), ^51^Zurek et al. (2011), ^52^Brophy et al. (2009).

**Figure 3:**
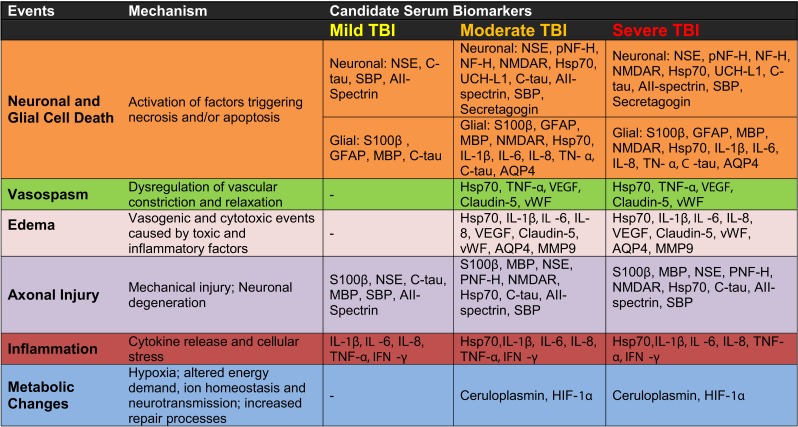
**Candidate serum protein biomarkers associated with injury severity and selected pathological mechanisms**.

Individuals who experience less than 30 minutes of lost or altered levels of consciousness after exposure to blast are classified as having suffered mild bTBI (mibTBI; Figure 1; Trudeau et al., 1998; Hoge et al., 2008; Elder et al., 2010; Levin et al., 2010; Rosenfeld and Ford, 2010; Wilk et al., 2010). This initial period may be followed by post-injury amnesia that lasts no longer than 24 hours. There are typically no penetrating injuries to the head or other organs, and neurological deficits (if any) are focal and transient in nature. Concurrently, the GCS score is nearly perfect at 13–15. The transient and mild neuropsychiatric deficits are typically followed by full recovery. However, similar to other types of mild TBI, a delayed onset of functional changes and long-term disabilities may still occur, especially after multiple exposures to mild blasts (Stern et al., 2011). Importantly, mibTBI shares symptoms and is often comorbid with post-traumatic stress disorder (PTSD; Hoge et al., 2008; Levin et al., 2010; Rosenfeld and Ford, 2010; Ursano et al., 2010). The combination of physical damage and psychological effects makes mibTBI especially difficult to diagnose. Thus, serum protein biomarkers that can distinguish between the physical and psychological components of the injury would be of special value (see also Figures 2 and 3 and discussion below).

Blast-induced traumatic brain injury is classified as moderate if loss of consciousness is longer than 30 minutes, post-injury amnesia lasts longer than 24 hours, and the initial GCS score is between 9 and 12 (Figure 1; Thompson et al., 2008; Aarabi and Simard, 2009; Wolf et al., 2009; Drake et al., 2010). The long-term outcome of moderate bTBI (mobTBI) can include detectable and significant levels of cognitive and neuropsychiatric abnormalities. Moderate bTBI can also be comorbid with PTSD and injuries to other organs.

According to the current classification system, bTBI is severe if the GCS score is less than 9 (Figure 1; Ling and Marshall, 2008; Ling et al., 2009; Ling and Ecklund, 2011). In the severe form of bTBI (sbTBI), polytrauma, i.e., injuries to other parts of the body, most frequently to the extremities, abdomen, and lungs can significantly contribute to and modify the pathology and outcome of brain injury. In addition to its comorbidity with polytrauma, sbTBI is often the result of multiple types of brain injury. Beyond the damage caused by the primary components of blast, various objects, debris, and shrapnel may break through the skull causing penetrating TBI. Also, it is not uncommon for the victim to be physically thrown from the mechanical force of explosion, causing further injury (acceleration-deceleration TBI). The combined damage from blast, penetrating injury, and acceleration-deceleration types of forces cause severe brain damage that leads to complex and debilitating long-term neurological and neuropsychiatric deficits, if not death (see Figures 2 and 3; Discussion below; Ling and Marshall, 2008; Ling et al., 2009; Ling and Ecklund, 2011). With respect to biomarkers, measuring changes in specific serum proteins indicative of the extent of neuronal and glial cell loss, axonal, and vascular damage, and damage to other organs can significantly add to the current diagnostic palette of neurobehavioral tests (see also Figures 2 and 3 and discussion below).

## Pathological Mechanisms and Associated Serum Protein Biomarkers in bTBI

The initial interactions between the physical forces of blast and the brain trigger pathological responses called the primary injury process or mechanism. The pathological components of the primary injury mechanism are largely influenced by whether the insult results in open (penetrating) or closed head injury. Penetrating head injury not only causes substantial direct tissue damage, but also instantaneously breaks down existing biological barriers, generating massive pathological responses to toxic molecules and cellular debris. In contrast, closed head injury typically causes metabolic changes and axonal damage of various degrees. In response to the typically short-lasting primary injury mechanism, there is a second wave of long-lasting pathological changes called the secondary injury mechanism. These pathologies include metabolic changes, neuroinflammation, axonal injury, vascular abnormalities, and neuronal and glial cell death (Ghirnikar et al., 1998; Lenzlinger et al., 2001; Vink et al., 2001; Morganti-Kossmann et al., 2002; Nortje and Menon, 2004; Warden et al., 2006; Cernak and Noble-Haeusslein, 2010; Donkin and Vink, 2010). Metabolic changes include abnormal levels of oxygenation (hypoxia), altered cell metabolism (e.g., glucose utilization), disrupted energy levels and utilization (leading to ionic imbalance, excitotoxicity, etc.), systemic hormonal secretion, and an upregulation of inflammatory activity (Cook et al., 2008; Feng et al., 2012). Inflammation is almost always a result of injury, and occurs in response to damaging stimuli, triggering the release and activation of cytokines and chemokines and the activation and proliferation of microglia (and astroglia) in the CNS. A propagating immune response may promote neurotoxicity and vascular changes (Morganti-Kossmann et al., 2007; Ziebell and Morganti-Kossmann, 2010; Brown et al., 2011). Vascular abnormalities are marked by aberrations in the water content of the brain parenchyma, dysregulation of water channels, and a compromised blood-brain barrier (BBB). Vascular abnormalities can be triggered by cyto- and vasogenic factors leading to edema, vasospasm, and altered rates of perfusion. Diffuse axonal injury is also common, and entails a loss of membrane integrity, altered axonal architecture, Wallerian-type axonal degeneration, metabolic disruption leading to degeneration, and increased serum levels of axonal proteins and filaments (Meythaler et al., 2001). Finally, neuronal and glial cell loss results from necrotic and apoptotic cell death during primary and secondary injury, and may lead to an increase in various neuron- and glia-specific proteins in serum (Stoica and Faden, 2010). Compounded together, these primary and secondary injury processes may lead to a range of neuropsychiatric symptoms, including various forms of memory and learning deficits, anxiety, and depression (Arciniegas, 2011).

**Figure 4:**
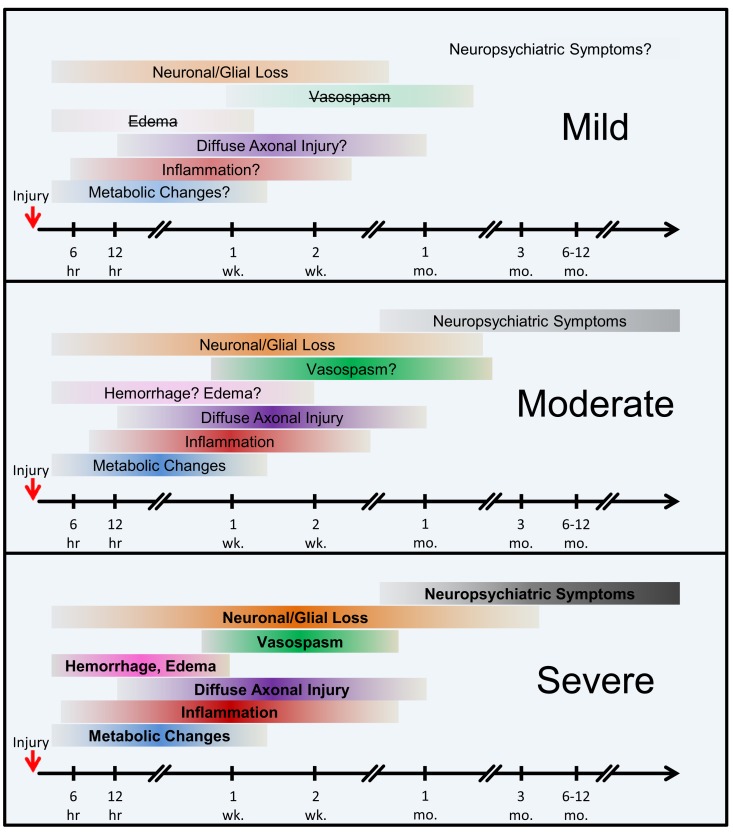
**The onset and the extent of selected pathological mechanisms of bTBI by injury severity. Hypothetical model**. The colored horizontal bars intend to illustrate the approximate onset and extent of the individual pathological changes following injury. The color intensities and bold type reflect an increased severity and contribution of the individual pathologies, whereas lighter colors, question marks, and strikethroughs represent weaker, relatively transient, or nearly absent conditions. Abbreviations: hr, hour(s); wk., week(s); mo., month(s).

According to our current understanding, the various forms of TBI can share common pathological “components” during both the primary and the secondary injury processes. What likely distinguishes the various forms of TBIs are the onsets and relative contributions of these individual components to the overall pathological cascades. Earlier works in different forms of TBI have identified candidate biomarkers associated with the various pathological changes (Figures 2 and 3). Many of these markers are neuron- and glia-specific and reflect damage to the different cellular components of the brain. Others are more ubiquitous and may indicate generalized metabolic changes, inflammation, etc. Changes in the serum levels of some of these markers have also been found in bTBI. The onset, intensity, and temporal patterns of the various pathological components likely depend on the severity of the injury (Figure 4). For example, one of the distinguishing features of sbTBI is the unusually early onset (within hours after exposure) and extent (high severity) of edema, whereas vasospasm is unusually delayed (up to 2 weeks post-injury; Ling and Marshall, 2008; Ling et al., 2009; Ling and Ecklund, 2011). It should also be noted that hemorrhage is not associated with mibTBI, but is highly characteristic of sbTBI (Ling and Marshall, 2008; Ling et al., 2009; Ling and Ecklund, 2011).

The temporal aspects of injury, like the onset of the various pathologies, are especially important in dynamically changing diseases like bTBI. An experimental study investigating the temporal pattern of changes in serum levels of four of the most commonly used clinical and experimental biomarkers, S100β, NSE, MBP, and NF-H showed that the temporal pattern of changes can reflect injury severity. Using a swine model of explosive blast and monitoring changes in the serum levels of the four markers up to 2 weeks post-injury, Gyorgy et al. (2011) found increases over time in the serum levels of all four markers. Importantly, the temporal pattern of changes in the serum levels of NF-H showed that in sbTBI, serum NF-H levels peaked early (within 6 hours after injury). The temporal pattern of changes of the other three markers showed no correlation with injury severity. This study illustrates how monitoring the temporal pattern of changes (e.g., “time to peak”) of serum biomarkers can be useful for identifying injury severity and outcome. This study also underlines the importance of monitoring changes in serum levels of several markers as they can reflect the dynamics of distinct but important pathologies, e.g., glial response (GFAP and S100β) vs. axonal damage (NF-H).

Experimental data derived from a rodent model of bTBI has shown that in addition to neuronal and glial cell damage, there are also vascular abnormalities that occur in mibTBI (Kovesdi et al., 2011; Kwon et al., 2011). Elevated serum levels of neuron- or glia-specific proteins (NF-H, NSE, CK-BB; GFAP, MBP, S100β) indicate increased permeability of the BBB (in addition to neuronal and glial cell damage or loss). In the same study, the authors found more direct evidence of vascular abnormalities. Serum levels of VEGF, a protein associated with regulating complex vascular functions including vascular permeability (Neufeld et al., 1999; Croll et al., 2004; Rosenstein and Krum, 2004), were significantly elevated. This study has the limitation of measuring serum levels of the protein markers only at a single, terminal time point. However, the terminal time point of the elevated serum protein markers was taken more than 2 months after injury. This finding indicates that there may be long-lasting ongoing pathological changes, even after a single exposure to mild blast. These findings can have great clinical relevance if they can be repeated. Given the large number of soldiers exposed to a single mild blast, and our very limited knowledge about the long-term consequences of blast, a longitudinal study focusing on a few protein biomarkers should be considered. The data from such a study can be correlated with long-term neurobehavioral assessments in order to identify individuals with increased vulnerabilities and also result in a better understanding of the pathobiology of mbTBI.

In addition to neuronal and glial damage, there is also experimental evidence of neuroinflammation and vascular changes as pathological responses to bTBI (Agoston et al., 2009; Kamnaksh et al., 2011; Kovesdi et al., 2011; Kwon et al., 2011). Along with elevated serum levels of VEGF, there is data showing increased levels of Claudin-5 and vWF in rodents after repeated exposures to mild blasts (Ahmed et al., in preparation). The temporal pattern of changes in their serum levels and the correlation between injury severity and the temporal patterns are currently being investigated (Ahmed et al., in preparation).

Evidence from the study discussed above also showed that animals that were exposed to stressful conditions (in order to simulate battlefield conditions and trigger PTSD) but were not injured had no increase in the serum levels of the previously mentioned protein biomarkers(Kwon et al., 2011). Despite this, they had increased serum corticosterone (CORT) levels and displayed behavioral pathologies like increased anxiety. These findings indicate that one may be able to design objective, serum-based differential diagnostics to distinguish between mibTBI and PTSD. Such a test would be especially important because miBTBI is the most frequent form of blast-induced neurotrauma, accounting for approximately 70% of all bTBIs (Trudeau et al., 1998; Thompson et al., 2008; Elder and Cristian, 2009; Elder et al., 2010; Rosenfeld and Ford, 2010). Moreover, PTSD has emerged as one of the most frequent and lasting consequences of recent military conflicts (Rosenfeld and Ford, 2010; Belanger et al., 2011; Luethcke et al., 2011). Mild bTBI and PTSD have overlapping neurobehavioral symptoms, clinically as well as experimentally. Based on current knowledge, exposure to blast can trigger cellular damage, thus requiring different therapeutic interventions than those used for PTSD (Thompson et al., 2008; Elder et al., 2010; Luethcke et al., 2011). Soldiers showing no functional deficit based on current neurobehavioral assessments (e.g., MACE) after exposure to mild levels of explosive blast return to duty and often become re-exposed to additional blasts (Hayes et al., 2012). As early studies implicate, additional exposures can have severe consequences, including an increased risk of developing long-term neuropsychiatric abnormalities (Okie, 2005; Peota, 2005; Aarabi and Simard, 2009; Jaffee and Meyer, 2009; Cernak et al., 2011; Plurad, 2011; Hayes et al., 2012). Serum protein biomarkers that can indicate the extent of individual vulnerability are of major value. For example, advanced “bio-dosimeters” can be developed for soldiers by using a combination of serum-based health information and physical parameters provided by helmet-mounted sensors (accelerometers). Such a personalized tool can indicate the real-time vulnerability of a soldier to any additional blast. Similar personalized “dosimeters” can also be developed for athletes with a high risk of repeated TBI, such as NFL players.

There are several conceptual, logistical, and technical problems associated with developing serum biomarkers as a diagnostic tool in neuronal insults like TBI. Technical problems include selecting the best proteomics method for serum biomarker discovery (Agoston et al., 2009). To facilitate the antibody-based validation of serum protein biomarkers in bTBI, we listed potential markers as a function of their association with different pathologies and severities of TBI (Figure 3). Some of the markers (“the usual suspects”) have been well studied, established, and analyzed in bTBI (Bauman et al., 2009; Gyorgy et al., 2011; Kovesdi et al., 2011; Kwon et al., 2011). Unfortunately, only a few of the listed markers have been verified by clinical studies to show changes in their serum levels specifically due to injury to the brain (and not to other organs). Even fewer markers have been evaluated in clinical settings and correlated with functional and neurobehavioral changes (e.g., GCS and other neurobehavioral tests) routinely used in clinical settings (Agoston et al., 2012).

In addition to aiding in the diagnosis and assessment of injury severity, serum protein biomarkers in mTBI can provide critical information for designing individualized treatment and for monitoring disease progression and treatment effectiveness. Simple versions of such an approach are already in use at neurointensive care units, where serum C-reactive Protein levels are monitored to assess general inflammation and treatment effectiveness. However, the current lack of clinical evidence about how changes in serum levels of protein biomarkers correlate to pathomechanisms and functional outcomes in TBI is a major hindrance. Concentrated, large scale, and preferably international research efforts are needed in order to generate reliable and clinically useful information for aiding in the evidence-based treatment of TBI.

## Summary and Future Directions

We would like to conclude this focused review by suggesting three relatively easy and implementable measures that can speed up both serum protein biomarker discovery and validation in bTBI. First: blood should be obtained at multiple time points (serial sampling) in both experimental and clinical studies in order to enable the longitudinal analysis of changes in serum levels of protein markers in bTBI. This approach can reveal temporal patterns of changes that may be of vital diagnostic and therapeutic value. Second: changes in the serum levels of a whole panel of proteins rather than a single protein need to be analyzed (at multiple time points). Multiplex assays, already in use in cancer biology, can provide substantially improved diagnostic precision, and especially so if combined with a systems biology analysis. Third: changes in serum protein biomarkers should be analyzed in relation to functional and neurobehavioral changes in both clinical and experimental settings. Such a combined analysis would radically improve the diagnostic and prognostic value of serum protein biomarkers by facilitating a much more direct understanding of how serum changes relate to functional deficits.

In addition to proteins, there are other potential biomarkers such as microRNAs. While microRNAs have some advantages (e.g., stability) compared to proteins, there is currently much less known about their functions. However, as our knowledge increases about their involvement in the various pathological processes their value as serum biomarkers will also increase.

In summary, serum-based protein biomarkers have the enormous potential to fundamentally change our understanding of bTBI and ultimately can – and will – be of major help in designing evidence-based treatments for individuals suffering from the consequences of blast injury.

## Conflict of Interest Statement

The authors declare that the research was conducted in the absence of any commercial or financial relationships that could be construed as a potential conflict of interest.

## Abbreviations

AQP4, aquaporin 4; C-tau, cleaved-tau protein; GFAP, glial fibrillary acidic protein; HIF-1α, hypoxia-inducible factor 1, alpha subunit; Hsp70, heat shock protein 70; IL-1β, interleukin-1 beta; IL-6, interleukin-6; IL-8, interleukin-8; MBP, myelin basic protein; MMP9, matrix metalloproteinase-9; NF-H, neurofilament-heavy chain; NSE, neuron specific enolase; pNF-H, phosphorylated neurofilament-heavy chain; S100β, S100 calcium binding protein B; SBPs, spectrin breakdown products; TNF-α, tumor necrosis factor alpha; UCH-L1, ubiquitin C-terminal hydrolase L1; VEGF, vascular endothelial growth factor; vWF, von Willebrand factor.
